# Cutaneous polyarteritis nodosa successfully treated with topical diflucortolone valerate – a case report & review of the literature

**DOI:** 10.1186/1546-0096-12-46

**Published:** 2014-10-10

**Authors:** Ruby Haviv, Maya Capua, Jacob Amir, Liora Harel

**Affiliations:** Department of Pediatrics C, Schneider Children’s Medical Center of Israel, Tel Aviv University, Sackler School of Medicine, Petach Tikvah, Israel; Department of Pediatrics Children’s Hospital at, Montefiore, New-York City, NY USA

**Keywords:** Cutaneous polyarteritis nodosa, Periarteritis, CPAN, Topical treatment, Corticosteroid, Diflucortolone valerate

## Abstract

Cutaneous Polyarteritis Nodosa (cPAN) was first described in 1931. cPAN is considered a rare disease, its true incidence is unknown. The age of onset is diverse. Most studies have shown no significant gender predominance. cPAN presents with distinct skin findings, such as a maculopapular rash, subcutaneous nodules, livedoid vasculitis, panniculitis, ischemic finger lesions, or erythematous patchy rash.

Etiology is unclear. It is still believed to be an immune complex-mediated disease, although a possible mechanism recently proposed relates a familial form of the disease to impaired activity of Adenosine Deaminase 2. cPAN may reflect an underlying disease, infection or medical treatment.

There is no consensus as to initial treatment, dosage and length of treatment. Patients with constitutional symptoms, visceral involvement, a more severe course of the disease, or high acute phase reactants, were treated mainly with systemic corticosteroids and/or cytotoxic agents for varying durations. However, persistence of cutaneous lesions has been documented.

We describe a 14 year old male suffering from persistent cPAN, with no constitutional symptoms or involvement of internal organs. The patient was treated with a local corticosteroid-based ointment during exacerbations, until complete remission. Although reported in only one study, treatment with topical corticosteroid compound may result in significant improvement or complete regression of skin lesions in cPAN patients.

## Background

The first description of limited cutaneous polyarteritis nodosa (cPAN) was published by Lindberg in 1931, describing skin findings, and also extra-cutaneous findings, such as fever, malaise, myalgia, arthralgia and neuropathy (unlike systemic PAN, in which the cutaneous findings are only secondary to internal organs involvement, mainly kidney, heart & liver) [1].

cPAN is rare; its true incidence is unknown. It is estimated that one third of children diagnosed with systemic PAN (sPAN), actually have cPAN [2, 3], but in practice, rheumatologists may treat more cPAN patients than sPAN patients.

Age of onset ranges from the neonatal and infantile period [4, 5], up to age 81 [6]. Most studies do not reveal any significant gender predominance [1]. A male to female ratio of 1:1.7 was found in a large study of 79 cases [6].

cPAN presents with distinct skin findings, such as a maculopapular rash, subcutaneous nodules, livedoid vasculitis, panniculitis, ischemic finger lesions, or erythematous patchy rash. In a study of juvenile polyarteritis, all patients with cPAN were diagnosed with necrotizing arterial inflammation found on biopsy [3].

The etiology of cPAN is unknown. It is, most probably, an immune complex-mediated disease, with some evidence of serum IgM anti-phosphatidylserine-prothrombin antibodies in patients’ sera, and deposition of C3 within vessel walls, as shown by direct immunofluorescence techniques [7]. Recently, loss-of-function mutations, in the gene (CECR1) encoding Adenosine Deaminase 2, were found to be related to a familial vasculopathy syndrome. Only one participant of Georgian ancestry in this study did not present with any cutaneous features, while visceral involvement was described in about half of the participants. The suggested mechanism is related to the chronically high levels of adenosine, or an impaired ADA2 function as a growth factor [5].

cPAN may reflect an underlying disease (ie inflammatory bowel disease [6]), infection (ie Hepatitis B virus, although findings were not consistent), or medications [1]. The most common agent identified is Group A β hemolytic Streptococcus.

There is no consensus as to initial treatment, dosage and length of treatment. However, in some studies, in which cPAN was found to be associated with a Streptococcal infection, prophylaxis with penicillin was initiated [1, 3, 8, 9]. Patients with constitutional symptoms, visceral involvement, a more severe course of the disease, or high acute phase reactants, were treated mainly with systemic corticosteroids, cyclophosphamide and/or azathioprine for varying durations [3]. If the patient was non-responsive, other studies reported IVIg [10, 11], colchicine, hydroxychloroquine, dapsone, methotrexate, sulphapyridine and pentoxifylline [1, 3, 6] as alternative treatments. Mild cases, consisting of mainly skin lesions, were treated with non-steroidal anti-inflammatory drugs or cholchicine. To date, only one case report investigating topical treatment for cPAN, among adult patients, has been published [12]. Persistence of cutaneous lesions has been documented. Rarely, did it progress to PAN.

## Case presentation

We present a 14 year old male, who had been suffering from cutaneous skin lesions, for 2 years prior to diagnosis. No other complaints or symptoms, such as fever, weight loss, arthritis, arthralgia, myalgia or hypertension were reported.

His past medical history was unremarkable, except for a milk allergy.

Family background: Both parents are Jewish, mother’s family from Eastern Europe; father’s family from Egypt. There were no reported rheumatologic or autoimmune diseases.

**Figure 1:**
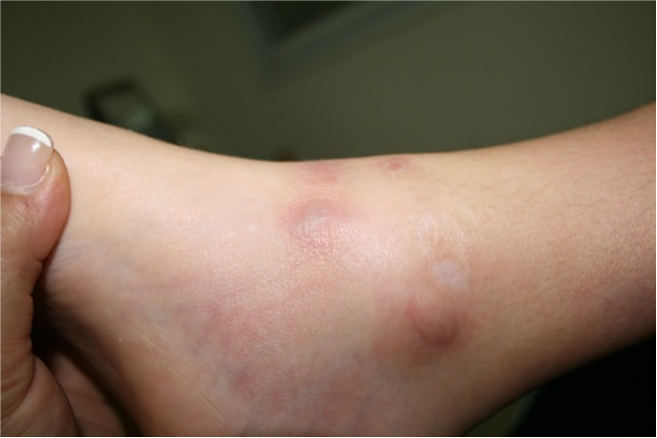
**A lower extremity nodular lesion.**

**Figure 2:**
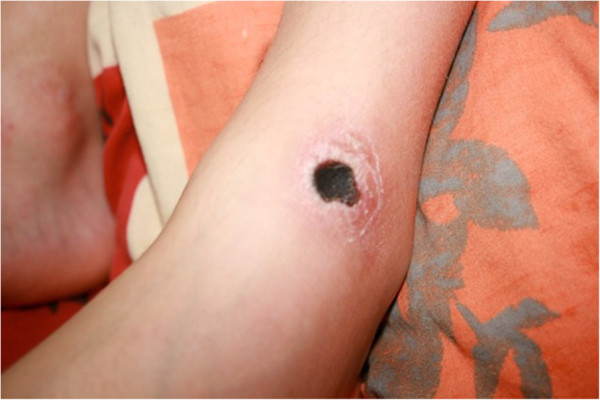
**The photos capture a lower extremity necrotic lesion in different stages of skin involvement.**

**Figure 3:**
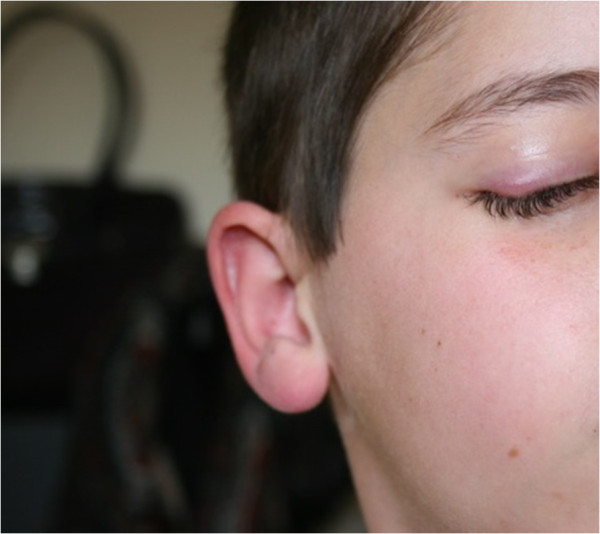
**The natural history of a right eyelid necrotizing lesion – early stage.**

**Figure 4:**
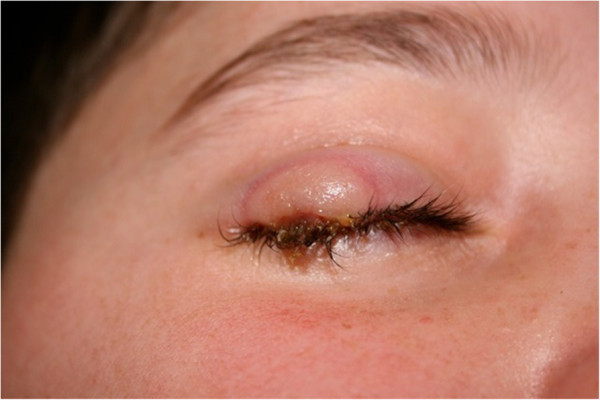
**The natural history of a right eyelid necrotizing lesion – progression.**

**Figure 5:**
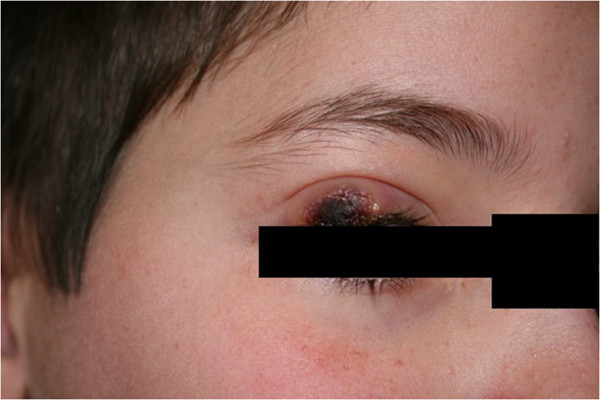
**The natural history of a right eyelid necrotizing lesion – necrosis.**

**Figure 6:**
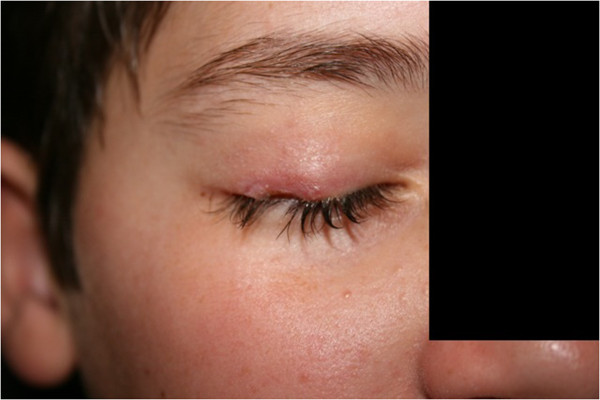
**The natural history of a right eyelid necrotizing lesion – healing.**

Physical examination was unremarkable, except for the nodular and tender skin lesions. The primary lesions were maculopapular, which had gradually evolved to tender and locally warm nodular skin lesions, later becoming necrotic. Trunk and extremities were involved (Figures 1, 2 capture lower extremity lesions in different stages of skin involvement), in addition to the patient’s face and right eyelid (Figures 3, 4, 5, 6 show the natural history of a right eyelid necrotizing lesion).

Laboratory workup, including acute phase reactants, complete blood count, liver and kidney function tests and urinalysis, were within normal ranges. Comprehensive infectious work-up was negative. Autoantibodies, including anti-nuclear antibodies, anti double-stranded DNA antibodies, and rheumatoid factor, were negative. Complement factors were normal. pANCA, cANCA, anti-myelperoxidase and anti-proteinase 3 antibodies, anti-Saccharomyces cerevisiae antibodies and antiphospholipid antibodies were all negative. Immunoglobulin profile was normal. Anti-Streptolysin O and anti-DNAse B antibodies were not detected. Hepatitis virus antibodies were negative. A whole blood sample was analyzed for common Familial Mediterranean Fever (FMF) mutations. The results were negative.

Abdominal ultrasound, including Doppler imaging of the renal arteries, chest x-ray and echocardiogram were all normal.

Skin biopsies were taken in another hospital, but the slides were re-examined in our hospital. Skin tissue samples showed widespread panniculitis and necrotizing medium vessel vasculitis, with fibrinoid necrosis, compatible with polyarteritis nodosa.

Since there were no indications of systemic involvement, based on the patient’s medical history, physical examination and laboratory results, he was diagnosed with cPAN.

Treatment with oral prednisone was recommended. Mainly concerned about the side effects of systemic steroidal treatment, the parents refused to any systemic treatment (including other oral agents, such as colchicine), relying on the boy’s good appearance and well-being. The patient was thus treated with topical diflucortolone valerate during exacerbations, three times a day, for two to three weeks at a time, until complete remission. Both facial and trunk lesions were treated, without any recurrence of a specific treated lesion reported during follow-up visits. If a lesion was not treated within days of its appearance, it would have become necrotic. No adverse reactions were reported, no extensive skin atrophy was marked, and no cataract was diagnosed upon follow-up. A lesion became infected only once (by Staphylococcus aureus and Pseudomonas aeruginosa), and was successfully treated with oral antibiotics.

## Conclusions

Currently, there is no consensus as to initial treatment, dosage and length of treatment in cPAN patients. We describe herein a 14 year old male, diagnosed with cPAN. Due to parental refusal to any systemic treatment, he was treated with a topical corticosteroid-based compound during exacerbations, applying the compound three times a day, for two to three weeks at a time. Treatment was very effective, resulting in a complete remission, without any recurrence or adverse effect documented during follow-up. If the compound had been applied after a few days delay, the lesion would have become necrotic. Topical corticosteroid compounds may be efficient in treating isolated skin lesions among cPAN patients, without mucosal or systemic signs of involvement. This is the first published case utilizing an effective topical treatment among juvenile patients. A prospective, multi-center study should be conducted, to evaluate different available treatments available, and weigh the different approaches in the light of the long-term adverse effects.

### Consent

Written informed consent was obtained from the patient’s parent for the publication of this report and any accompanying images.
